# RRx-001 inhibits G6PD to deplete NADPH and trigger disulfidptosis coupled with DAMP-mediated immunogenic cell death in hepatocellular carcinoma

**DOI:** 10.1038/s41420-026-03032-y

**Published:** 2026-03-26

**Authors:** Hailian Huang, Yongfei He, Jingxuan Chen, Yuan Liao, Shutian Mo, Wei Qin, Meifeng Chen, Tianyi Liang, Guohong Yan, Shuxin Wei, Qichong Xie, Xiaoling Luo, Chuangye Han

**Affiliations:** 1 School of Basic Medical Sciences, Guangxi Medical University, Nanning, China; 2 Department of Pathology, The Second Affiliated Hospital of Guangxi University of Science and Technology, Liuzhou, China; 3 Key Laboratory of Early Prevention and Treatment for Regional High Frequency Tumor, Ministry of Education, Guangxi Medical University, Nanning, China; 4 Department of Hepatobiliary Surgery, The First Affiliated Hospital of Guangxi Medical University, Nanning, China; 5 Department of Hepatobiliary and Pancreatic Surgery, The First Affiliated Hospital of Zhengzhou University, Zhengzhou, China; 6 Department of Hepatobiliary, Pancreatic and Vascular Surgery, The Second Affiliated Hospital of Guangxi Medical University, Nanning, China; 7 Guangxi Key Laboratory of Enhanced Recovery After Surgery for Gastrointestinal Cancer, Nanning, China

**Keywords:** Cell death, Tumour immunology

## Abstract

Disulfidptosis is a recently identified form of programmed cell death driven by NADPH metabolic imbalance and disulfide stress, but its therapeutic relevance in hepatocellular carcinoma (HCC) remains poorly understood. RRx-001, a clinical-stage small-molecule agent known for its epigenetic modulatory and radiosensitizing effects, has yet to be explored for its potential to induce disulfidptosis and immunogenic cell death (ICD). This study investigated the mechanism by which RRx-001 triggers disulfidptosis in HCC through NADPH metabolic dysregulation and evaluated its capacity to elicit ICD and antitumor immunity. Using Huh-7 and Hepa1-6 HCC cell lines and a murine subcutaneous xenograft model, we assessed drug sensitivity (CCK-8), apoptosis (flow cytometry), metabolic parameters (NADPH, GSH/GSSG, ROS), and ultrastructural changes (transmission electron microscopy). Protein expression was analyzed by immunofluorescence and Western blotting. In vivo antitumor efficacy was evaluated, and immune microenvironment dynamics were characterized via transcriptomic sequencing, immunohistochemistry, and flow cytometry, with all animal experiments randomized and blinded. RRx-001 markedly reduced NADPH levels by downregulating G6PD, leading to redox imbalance (decreased GSH/GSSG ratio, elevated ROS) and F-actin cytoskeletal contraction—hallmarks of disulfidptosis. This process was partially reversed by the disulfide reductant TCEP, confirming disulfidptosis dependency; quantitative F-actin fluorescence intensity showed significant contraction in RRx-001-treated cells that was mitigated by TCEP co-treatment (*p* < 0.01). Additionally, RRx-001 promoted the release of damage-associated molecular patterns (DAMPs), including CRT, HMGB1, and HSP70/90, activating ICD, as confirmed by ELISA of extracellular HSP70/90. In vivo, RRx-001 significantly suppressed tumor growth (*p* < 0.001), reduced tumor weight, enhanced infiltration of CD4+ and CD8+T cells, increased M1 macrophage polarization, and downregulated PD-L1 expression. Transcriptomic analysis implicated enhanced antitumor immunity via T-cell receptor signaling and T-helper cell differentiation pathways. These findings demonstrate that RRx-001 triggers disulfidptosis in HCC by targeting the G6PD–NADPH axis while concurrently inducing ICD, achieving dual metabolic and immunomodulatory effects. This mechanistic insight provides a scientific foundation for developing novel disulfidptosis-based HCC therapies with high translational potential and suggests that future studies should explore the synergistic efficacy of RRx-001 with immune checkpoint inhibitors.

**Figure:**
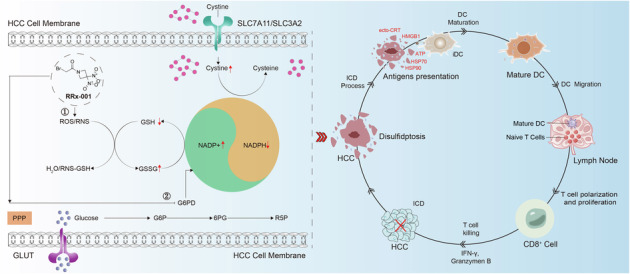
This study uncovers the therapeutic mechanism of RRx-001 in hepatocellular carcinoma (HCC): by inhibiting G6PD, it reduces NADPH levels, leading to redox imbalance and F-actin contraction, thereby inducing disulfidptosis—a process that can be partially reversed by TCEP. Meanwhile, RRx-001 promotes the release of DAMPs, activating immunogenic cell death (ICD). In vivo, RRx-001 significantly inhibits tumor growth, enhances T-cell infiltration, promotes M1 macrophage polarization, downregulates PD-L1 expression, and strengthens anti-tumor immunity through T cell-related pathways. With both metabolic and immunomodulatory effects, RRx-001 provides a basis for novel HCC therapies, and future research could explore its synergistic effects with immune checkpoint inhibitors.

This study uncovers the therapeutic mechanism of RRx-001 in hepatocellular carcinoma (HCC): by inhibiting G6PD, it reduces NADPH levels, leading to redox imbalance and F-actin contraction, thereby inducing disulfidptosis—a process that can be partially reversed by TCEP. Meanwhile, RRx-001 promotes the release of DAMPs, activating immunogenic cell death (ICD). In vivo, RRx-001 significantly inhibits tumor growth, enhances T-cell infiltration, promotes M1 macrophage polarization, downregulates PD-L1 expression, and strengthens anti-tumor immunity through T cell-related pathways. With both metabolic and immunomodulatory effects, RRx-001 provides a basis for novel HCC therapies, and future research could explore its synergistic effects with immune checkpoint inhibitors.

## Introduction

Hepatocellular carcinoma (HCC) represents the most prevalent type of liver cancer, carrying a substantial global disease burden. Despite continuous advancements in HCC diagnosis and treatment techniques, including surgical resection, liver transplantation, local ablation therapy, and the clinical application of molecularly targeted drugs, the overall survival rate of patients remains unsatisfactory, with a 5-year survival rate of less than 20% [1, 2]. This predicament primarily stems from the high heterogeneity of HCC, difficulties in early diagnosis, susceptibility to metastasis and recurrence, and drug resistance to systemic therapies [3, 4]. In recent years, developing novel cell death models has opened up promising new pathways for the preclinical and clinical treatment of HCC. For instance, some researchers have discovered that combining low-dose oxaliplatin with leflunomide, a dihydroorotate dehydrogenase (DHODH) inhibitor, can suppress the progression of HCC by inducing ferroptosis. This approach significantly enhances the sensitivity of chemotherapy and reduces toxic reactions associated with it [5]. Simultaneously, other studies have elucidated the mechanistic role of proteasome inhibitors in triggering pyroptosis in HCC, confirming that inhibiting proteasome activity and WEE family kinases represents a highly potential novel anticancer therapy strategy targeting solid tumor cells [6]. Therefore, exploring new therapeutic targets and mechanisms, particularly strategies based on tumor metabolic reprogramming and immune microenvironment modulation, has become the focus of current research.

In recent years, the diversity and regulatory mechanisms of programmed cell death (PCD) have garnered significant attention. Beyond the classical forms of apoptosis and necrosis, novel modes of cell death such as ferroptosis and cuproptosis have been discovered, offering new avenues for tumor treatment [7, 8]. Among these, disulfidptosis, a newly identified type of PCD, stands out due to its unique metabolic dependency and potential clinical applications. The core mechanism of disulfidptosis involves the imbalance of intracellular cystine/cysteine metabolism, leading to disulfide bond stress. Under glucose starvation, the insufficient supply of NADPH impairs the effective reduction of cystine to cysteine, resulting in abnormal accumulation of disulfides. This, in turn, triggers rapid cell death through the formation of non-physiological disulfide bonds in the F-actin cytoskeleton [9]. Studies have indicated that high expression of SLC7A11 (cystine/glutamate antiporter) increases tumor cells’ dependency on NADPH, making them more susceptible to disulfidptosis under glucose deprivation or oxidative stress conditions [9, 10]. This mechanism has shown therapeutic potential in various solid tumors, including bladder cancer, ovarian cancer, and glioma, but research on HCC is still in its infancy [11–13]. Although bioinformatics-based studies have initially untangled the molecular markers of disulfidptosis in HCC, the validity and accuracy of these markers remain to be verified [14, 15]. Most importantly, the current clinical landscape lacks effective therapeutic agents targeting disulfidptosis, significantly limiting the application potential of disulfidptosis theory in clinical practice.

NADPH, as a crucial intracellular reducing power molecule, serves as a central factor in maintaining redox homeostasis and resisting oxidative stress. Its depletion can induce disulfide stress, subsequently triggering disulfidptosis. The generation pathways of NADPH primarily include the pentose phosphate pathway (PPP), folate metabolism, fatty acid oxidation, and glutamine catabolism [16, 17]. Among these, PPP contributes approximately 60–80% of NADPH production, with glucose-6-phosphate dehydrogenase (G6PD) as the rate-limiting enzyme of PPP. The activity of G6PD directly determines the efficiency of NADPH synthesis [16]. Inhibiting G6PD can deplete NADPH, disrupt the glutathione (GSH) system, and induce oxidative stress and cell death. This strategy has demonstrated antitumor effects in various tumor models [18, 19]. However, traditional intervention methods, such as glucose deprivation or exogenous H₂O₂ treatment, face challenges in translational application due to their lack of targeting specificity and clinical safety concerns [20]. Therefore, the current research focus is on developing small molecule compounds that specifically inhibit NADPH metabolic pathways, particularly targeting the PPP. This intervention strategy holds promise as a new approach to induce disulfidptosis and treat related diseases.

This study explored the mechanism by which RRx-001 reduces NADPH production by inhibiting G6PD activity and induces a large amount of ROS/RNS, thereby triggering disulfide death in HCC. Experimental results demonstrate that RRx-001-induced NADPH depletion leads to redox imbalance, manifested by a decreased glutathione (GSH)/oxidized glutathione (GSSG) ratio, elevated ROS levels, and F-actin contraction. Furthermore, RRx-001 promotes the release of DAMPs, such as CRT and HMGB1, thereby activating ICD. In vivo experiments confirm that RRx-001 significantly inhibits tumor growth while enhancing CD4+/CD8+ T cell infiltration and M1 macrophage polarization. Transcriptomic analysis reveals its regulatory effects on immune-related pathways. This research untangles the dual metabolic-immunological mechanism underlying RRx-001’s anti-HCC effects, providing a theoretical foundation for novel therapeutic strategies based on disulfidptosis and ICD.

## Results

### Exploring the triggering mechanism of disulfidptosis in hepatocellular carcinoma based on the NADPH metabolic pathway

**Fig. 1:**
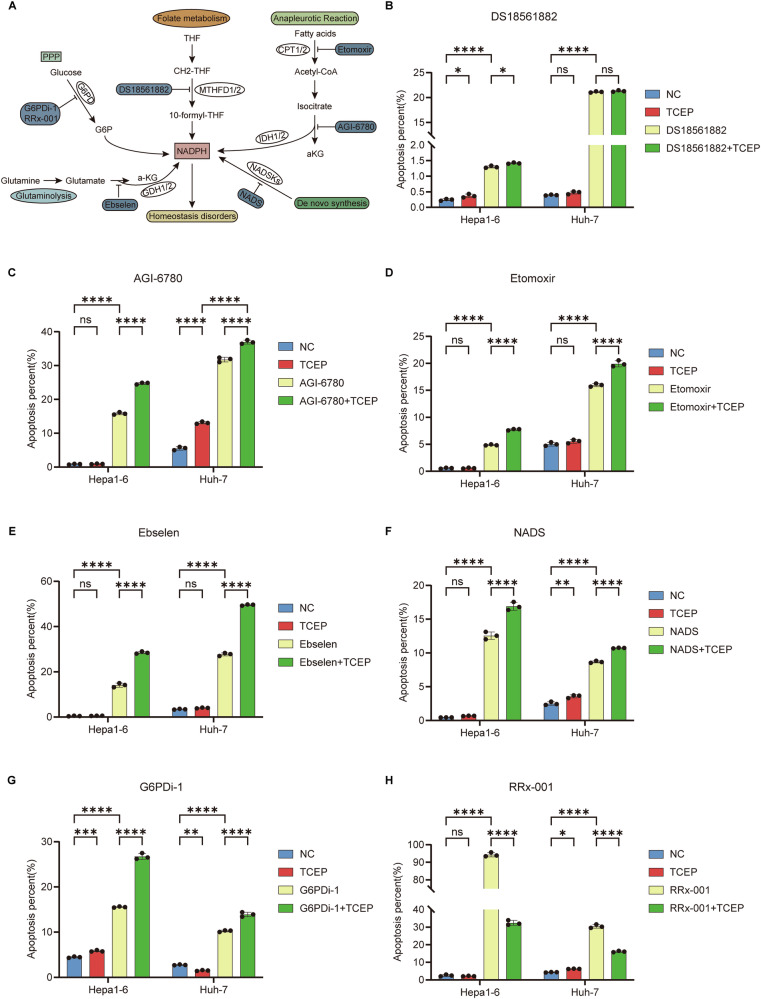
Explores the triggering mode of disulfide death in hepatocellular carcinoma based on the NADPH metabolic pathway. **A** NADPH generation pathway and corresponding inhibitors. **B**–**H** the effects of different compounds alone or in combination with TCEP on apoptosis. **B** folic acid metabolic pathway inhibitor (DS18561882). **C** isocitrate metabolic pathway (AGI-6780). **D** Fatty acid metabolic pathway (Etomoxir). **E** glutamine metabolic Pathway (Ebselen). **F** de novo Synthesis Pathway (NADS). **G** PPP approach (G6PDi-1). **H** PPP approach (RRx-001). ns No significant difference. *P* > 0.05; *P < 0.05, **P < 0.01, ***P < 0.001, ****P < 0.0001.

We first conducted a literature search to identify the major NADPH-generating pathways and their corresponding inhibitors, including inhibitors of the folate metabolism pathway (DS18561882), isocitrate metabolism pathway (AGI-6780), fatty acid metabolism pathway (Etomoxir), glutamine metabolism pathway (Ebselen), de novo synthesis pathway (NADS), and the pentose phosphate pathway (PPP) (G6PDi-1 and RRx-001) (Fig. 1A). To further validate the specific induction of disulfidptosis by RRx-001, we performed rescue experiments using TCEP, a disulfide bond reducer.Subsequently, we determined the half-maximal inhibitory concentration (IC50) of each inhibitor in hepatocellular carcinoma (HCC) cells using the CCK-8 assay to establish the experimental concentrations for subsequent studies. RRx-001 exhibited the lowest IC50 values (5.30–6.70 μM) among the inhibitors targeting various NADPH-generating pathways, including the PPP, folate metabolism, fatty acid oxidation, and glutamine deamination, significantly lower than those of other inhibitors (Fig. S1).

TCEP, a disulfide bond reducing agent, can effectively inhibit disulfidptosis. We added TCEP to the DS18561882, AGI-6780, Etomoxir, Ebselen, NADS, G6PDi-1, and RRx-001 groups and detected cell apoptosis by flow cytometry. The results showed that TCEP failed to reverse the apoptosis induced by DS18561882, AGI-6780, Etomoxir, Ebselen, NADS, and G6PDi-1. However, in the RRx-001 group, the application of TCEP partially reversed the apoptosis induced by RRx-001 (Fig. S2, Fig. 1B–H). These results suggest that RRx-001 is highly effective and may be the optimal small-molecule drug for triggering disulfidptosis.

### RRx-001 induces disulfidptosis in HCC cells via the G6PD/NADPH/ROS

**Fig. 2:**
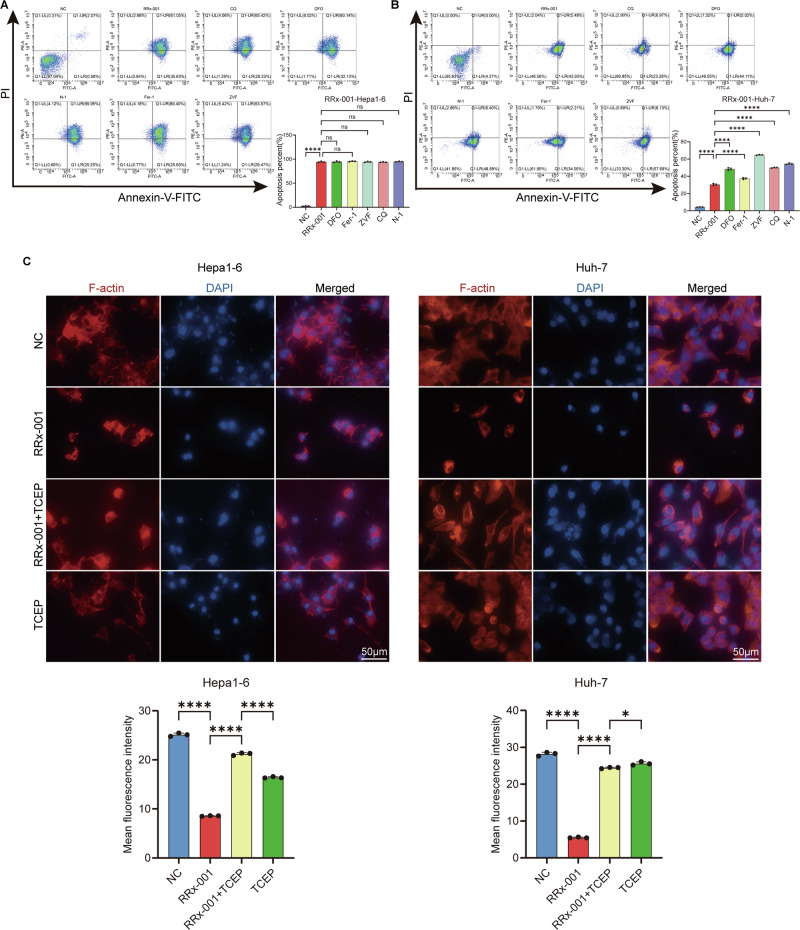
RRx-001 induces disulfide death in HCC cells. **A** apoptosis of Hepa1-6 cells under different treatment conditions. **B** apoptosis of Huh-7 cells under different treatment conditions. **C** immunofluorescence images of F-actin protein in hepatoma cells intervened by RRx-001 alone or in combination with TCEP. F-actin was labeled with red fluorescence (F-actin), the cell nucleus was labeled with blue fluorescence (DAPI), and Merge was the merged image. Quantitative analysis of F-actin fluorescence intensity is provided. Data are presented as mean ± SD; ns not significant difference. *P* > 0.05; ****P < 0.0001. The image method magnification is 400 times. ns No significant difference. *P* > 0.05; ****P < 0.0001.

To further verify that RRx-001 can induce disulfidptosis, we intervened with cells using RRx-001 and inhibitors of other types of cell death, such as ferroptosis, autophagy, and necroptosis. All inhibitors were administered 1 h prior to RRx-001 treatment.The results showed that RRx-001 induced apoptosis, and there was no significant difference compared with other inhibitors such as ferroptosis and necroptosis inhibitors (Fig. 2A, B). Quantitative analysis of F-actin fluorescence intensity revealed significant contraction in RRx-001-treated cells, which was partially reversed by TCEP co-treatment (*p* < 0.01). Further observation of cell morphology revealed that after RRx-001 treatment, the F-Actin-regulated cytoskeleton significantly contracted and separated from the cell membrane (Fig. 2C), consistent with the typical morphological characteristics of disulfidptosis. Western blot analysis confirmed that RRx-001 treatment significantly downregulated G6PD protein expression in a dose-dependent manner (Fig. S3). Knockdown of SLC7A11 using shRNA attenuated RRx-001-induced cell death, supporting its essential role in disulfidptosis (Fig. S4).

**Fig. 3:**
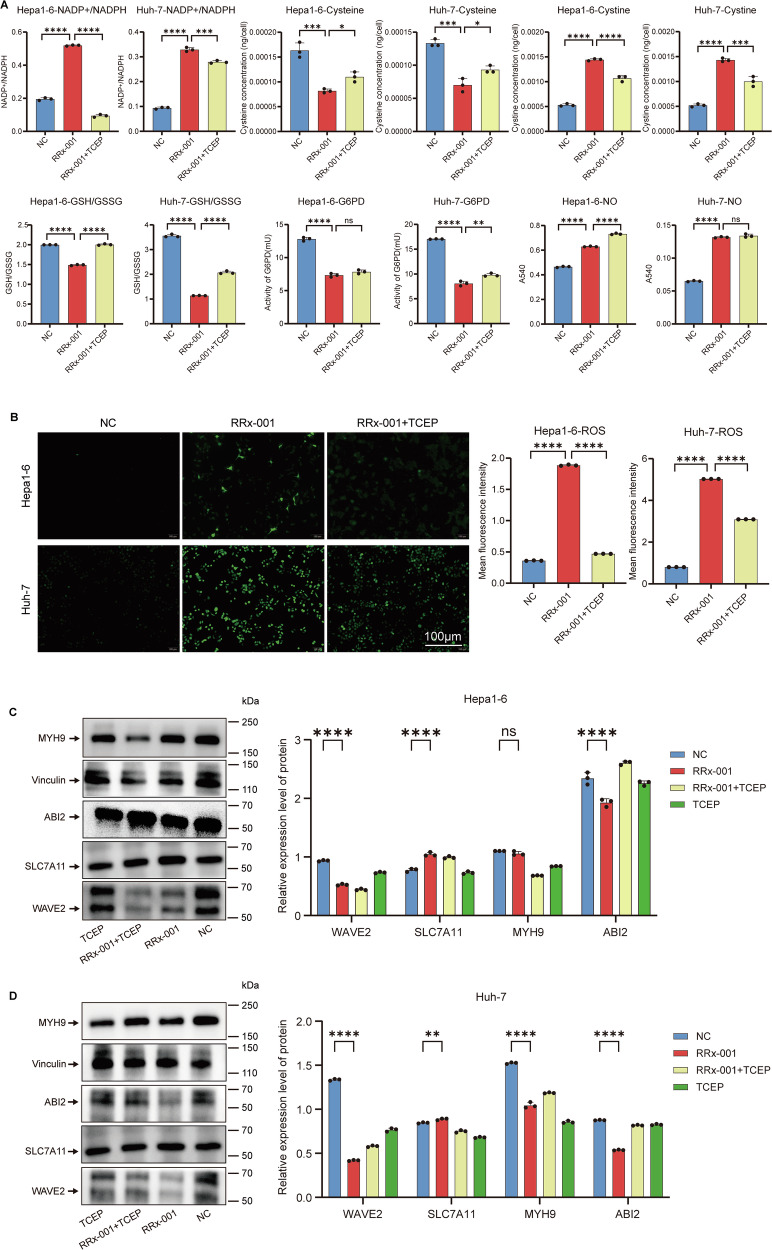
RRx-001 induces disulfide death in HCC cells. **A** the changes of metabolism-related indicators of hepatoma cells under the intervention of RRx-001 alone or in combination with TCEP. It includes indicators such as NADP+/NADPH, Cysteine, Cystine, GSH/GSSG ratio, G6PDH activity, and NO concentration. **B** the changes of ROS in hepatoma cells under the intervention of RRx-001 alone or in combination with TCEP, with a scale of 100 μm. **C**, **D**, on the left is the result graph of the protein expressions of WAVE2, SLC7A11, MYH9 and ABI2 in hepatoma cells (Hepa1-6 and Huh-7) of different treatment groups detected by Western blot, and on the right is the bar graph of the relative expression levels of the corresponding proteins. ns No significant difference. *P* > 0.05; *P < 0.05, **P < 0.01, ***P < 0.001, ****P < 0.0001.

G6PD is the sole rate-limiting enzyme in the pentose phosphate pathway (PPP). Rapidly proliferating cells require metabolites from the PPP to synthesize ribonucleotides and maintain intracellular redox homeostasis. Therefore, we detected NADP+/NADPH, Cysteine, Cystine, glutathione (GSH)/oxidized glutathione (GSSG), glucose-6-phosphate dehydrogenase (G6PDH), and nitric oxide (NO). The results showed that G6PD inhibition led to reduced NADPH production, an increased intracellular NADP+/NADPH ratio, decreased Cysteine levels, increased Cystine levels, and a decreased GSH/GSSG ratio. This indicated a severe depletion of the cell’s reducing capacity, accompanied by NADPH exhaustion, increased NO levels, and partial reversal by TCEP (Fig. 3A). Similar trends were observed in the detection of cellular reactive oxygen species (ROS) (Fig. 3B). Further Western blotting showed downregulation of actin regulatory proteins WAVE2 and MYH9, downregulation of ABI2, and upregulation of SLC7A11, suggesting abnormal disulfide bond formation in the cytoskeletal network (Fig. 3C, D). The inconsistent expression trends of MYH9 and ABI2 in Hepa1-6 cells may be attributed to cell type-specific regulatory mechanisms or feedback responses to disulfide stress. In summary, we preliminarily believe that RRx-001 induces disulfidptosis in HCC cells via G6PD.

**Fig. 4:**
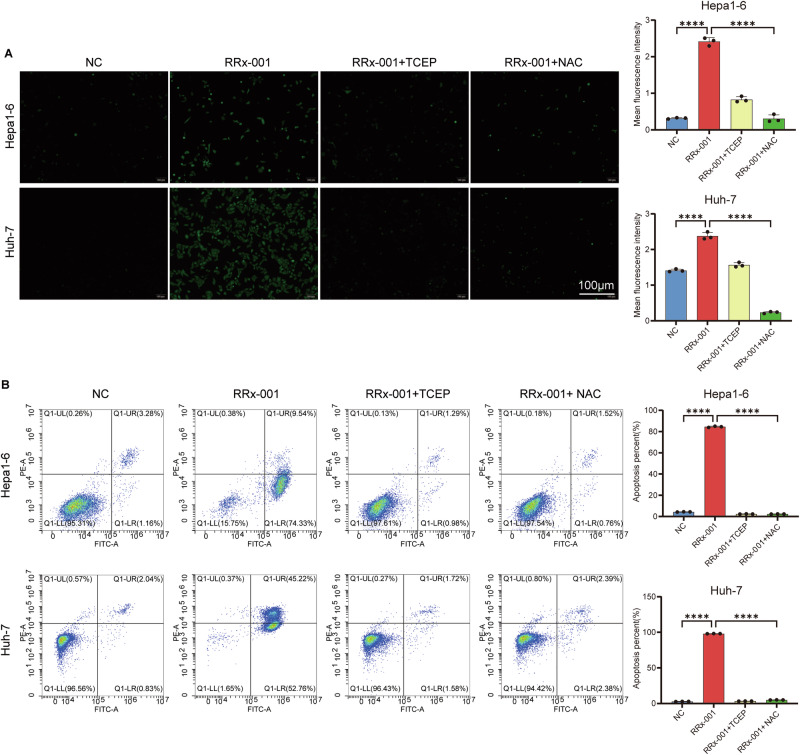
The association between RRx-001 induced cell death and ROS. **A** the changes of ROS after the intervention of RRx-001 alone or in combination with ROS scavengers. The left side shows the images of ROS levels in hepatoma cells of different treatment groups observed by fluorescence microscopy, with green fluorescence representing ROS and a scale of 100 μm. The right side shows the bar chart of the average fluorescence intensity of ROS in the cells of the corresponding treatment group. **B** apoptosis after the intervention of RRx-001 alone or in combination with ROS scavengers. On the left is the scatter plot of the apoptosis of hepatoma cells in different treatment groups detected by flow cytometry, and on the right is the bar plot of the apoptosis rate of the corresponding treatment groups. ns No significant difference. *P* > 0.05; *P < 0.05, **P < 0.01, ***P < 0.001, ****P < 0.0001.

ROS are closely related to disulfidptosis. ROS promote the abnormal accumulation of cystine within cells by inducing disulfide stress and disrupting protein function, thereby triggering actin cytoskeletal contraction and cell death.To investigate whether RRx-001-induced cell death is associated with ROS production, we conducted ROS scavenging experiments. The results showed that compared with RRx-001 treatment alone, ROS scavengers effectively reduced the elevated ROS levels induced by RRx-001 (Fig. 4A). Further comparison revealed that compared with the RRx-001 treatment group alone, the cell apoptosis in the RRx-001 combined with ROS scavenger group and the RRx-001 combined with TCEP group was reversed (Fig. 4B). These results indicate that ROS scavengers can effectively reduce the elevated ROS levels induced by RRx-001.

### RRx-001 triggers immunogenic cell death via DAMP release

**Fig. 5:**
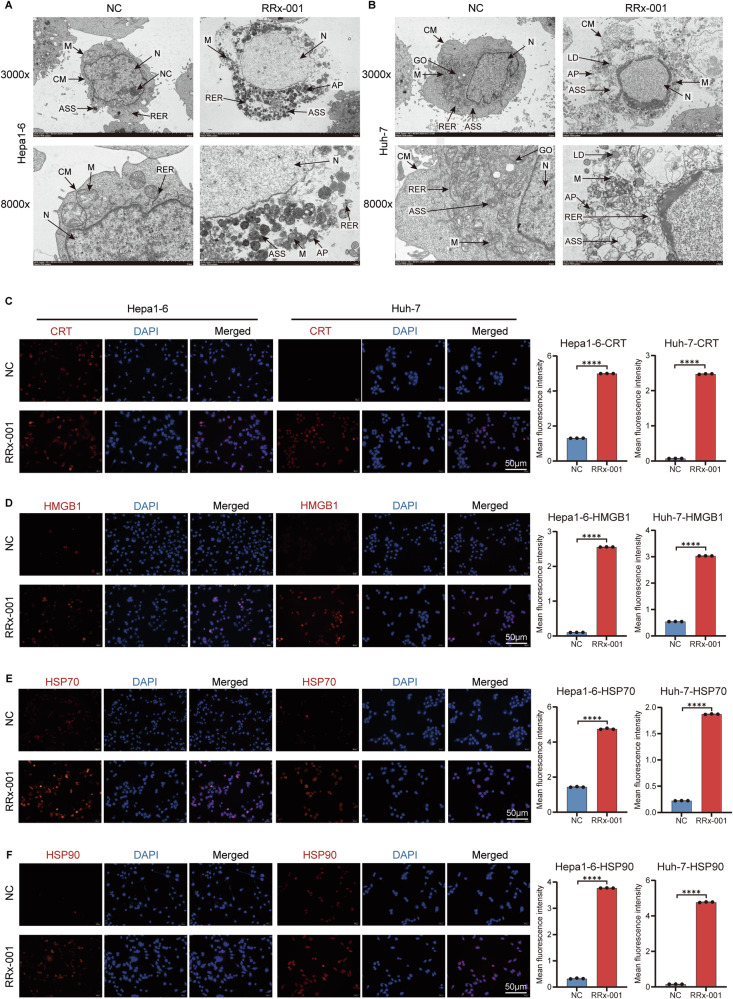
RRx-001 triggers immunogenic cell death through the release of DAMP. **A**, **B** The ultrastructure of hepatoma cells in different treatment groups was observed by transmission electron microscopy. The magnifications of the images are 3000 times and 8000 times respectively. **C**–**F** immunofluorescence staining was used to detect the release of DAMP in hepatoma cells of different treatment groups, including CRT, HMGB1, HSP70 and HSP90. On the left is the immunofluorescence image, with the target proteins (CRT, HMGB1, HSP70, HSP90) labeled in red fluorescence and the cell nucleus (DAPI) labeled in blue fluorescence. The Merge is the combined image, with a scale of 50μm. On the right is the bar chart of the average fluorescence intensity of the corresponding proteins. N Nucleus, CM Cell Membrane, M Mitochondrion, AP Primary Lysosome, LD Lipid Droplet, GO Golgi Apparatus, RER Rough Endoplasmic Reticulum, ASS Acinar-related Structure, ns No significant difference. *P* > 0.05; *P < 0.05, **P < 0.01, ***P < 0.001, ****P < 0.0001.

To further explore the effects of RRx-001 intervention on cells, we used electron microscopy to observe cells treated with RRx-001, focusing on cell surface morphology, cell membrane structure, and the shape, size, and distribution of various intracellular organelles. Under the intervention of RRx-001, both Hepa1-6 and Huh-7 cells exhibited significant changes. Specifically, Hepa1-6 cells showed an irregular overall morphology, obvious internal structural changes, abnormal distribution of cytoplasmic components, mitochondrial swelling with disrupted cristae, altered morphology of the rough endoplasmic reticulum and ribosome attachment, changes in autophagy-related structures, and varying degrees of changes in the nucleus and cell membrane. Huh-7 cells showed a significant change in overall morphology, uneven distribution and disordered structure of cytoplasmic components, mitochondrial vacuolization with reduced cristae, expansion or abnormal morphology of the rough endoplasmic reticulum, changes in autophagy-related structures, and corresponding changes in organelles such as the Golgi apparatus, lipid droplets, and autophagosomes. In addition, RRx-001 may also affect the redox balance and metabolic pathways of Huh-7 cells, regulate the autophagy and apoptosis processes of the cells, and change the expression of cell surface molecules (Fig. 5A, B). These cellular changes are consistent with some characteristics of immunogenic cell death, but further verification is needed.

Therefore, to observe the changes in DAMPs after RRx-001 intervention, we performed immunofluorescence analysis on cells treated with RRx-001. The results showed that after RRx-001 treatment, the expression levels of CRT, HSP70, HSP90 and HMGB1 in the cells all increased (Fig. 5C–F). ELISA and western blot analysis of cell confirmed the extracellular release of HSP70 and HSP90 following RRx-001 treatment (Fig. S5). These results further confirm that RRx-001-induced disulfidptosis has the effect of inducing ICD.

### RRx-001 inhibits tumor growth and enhances anti-tumor immunity in vivo

**Fig. 6:**
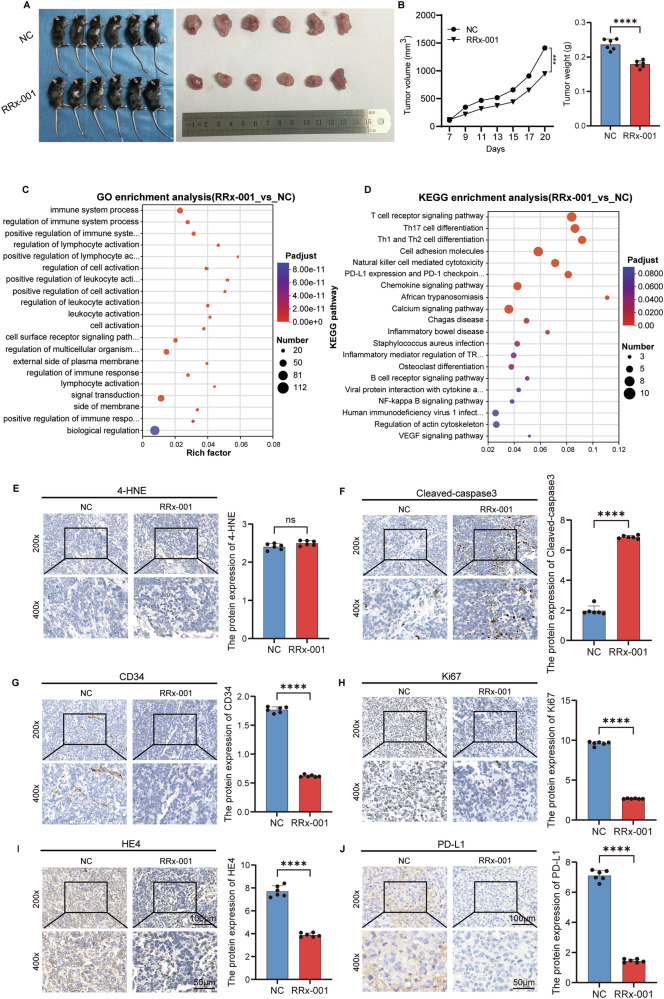
Research on the effect of RRx-001 on tumor growth and related molecular mechanisms. **A** Comparison of tumor appearance and size between the control group (NC) and the RRx-001 treatment group of mice. On the left is a photo of a mouse, and on the right is a photo of the exfoliated tumor tissue. **B** tumor growth curve, changes in tumor volume of mice in the control group (NC) and the RRx-001 treatment group within 7–20 days. Tumor weight was also measured and statistically analyzed. Data are presented as mean ± SD; *P < 0.05, *P* < 0.01. **C** Gene Ontology (GO) enrichment analysis, comparing the items related to biological processes, cellular components and molecular functions enriched in the RRx-001 treatment group and the control group. **D** Kyoto Encyclopedia of Genes and Genomes (KEGG) enrichment analysis, comparing the RRx-001 treatment group with the control group to show the enriched signaling pathways. **E**–**J** Immunohistochemistry was used to detect the protein expression of 4-HNE, CD34, HE4, Cleaved-caspase3, Ki67 and PD-L1 in tumor tissues of the control group and the RRx-001 treatment group, scale bars: 200× and 400× as indicated. On the left are the immunohistochemical staining images, with scales of 200 times and 400 times. On the right is the bar chart of the quantitative analysis of the corresponding protein expression. ns, No significant difference. *P* > 0.05; *P < 0.05, **P < 0.01, ***P < 0.001, ****P < 0.0001.

To evaluate the therapeutic effect of RRx-001 on HCC, we established a subcutaneous HCC xenograft model in mice and conducted intratumoral treatment. The results showed that compared with the NC group, tumor growth in the RRx-001 treatment group was significantly inhibited (Fig. 6A, B). Tumor weight was significantly reduced in the RRx-001 treatment group compared to controls (*p* < 0.001, Fig. 6B). To further explore the mechanisms underlying RRx-001-induced ICD, we performed transcriptome sequencing analysis on tumor tissues from the NC and RRx-001 groups. GO and KEGG enrichment analysis results showed that the differentially expressed genes were closely related to biological processes such as immune system processes, regulation of immune system processes, and signal transduction, and were significantly enriched in signaling pathways such as T cell receptor, PD-L1 expression and PD-1 checkpoint, and Th17, Th1, and Th2 cell differentiation (Fig. 6C, D). Immunohistochemical detection results showed that after RRx-001 treatment, the expression level of Cleaved-caspase3 in the tumor increased, while the expression levels of CD34, Ki67, HE4, and PD-L1 decreased (Fig. 6E–J). All immunohistochemical images include scale bars (200× and 400× magnification).

**Fig. 7:**
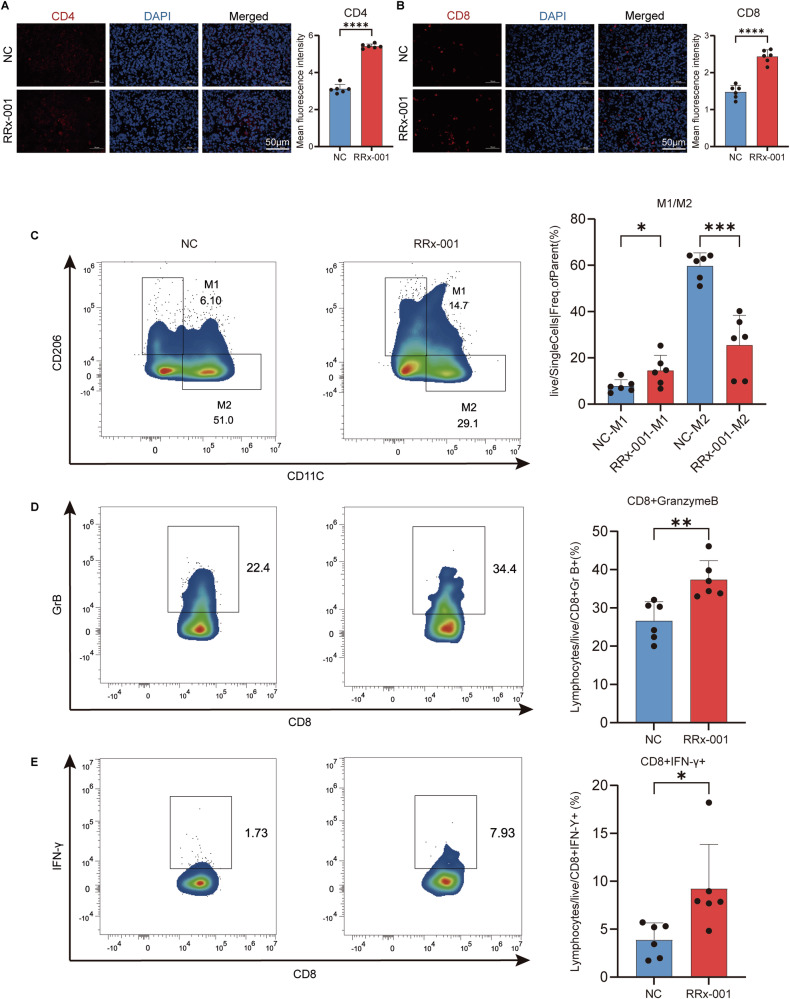
The effect of RRx-001 on immune cell subsets in the tumor microenvironment. **A**, **B** immunofluorescence of CD4+ and CD8+ T cells in tumor tissues. On the left are the fluorescence images of CD4+ (red) and CD8+, DAPI (blue, labeled with the cell nucleus), and Merge (merge), respectively, with A scale of 50μm. On the right is the bar chart of the average fluorescence intensity of CD4+ and CD8+, with a scale of 50 μm. **C**–**E** Flow cytometry was used to analyze the proportions of different immune cell subsets in the tumor tissues of the control group (NC) and the RRx-001 treatment group. From top to bottom, they were M1 and M2 type macrophages, CD8+ Granzyme B and CD8+IFN-γ+ cells, and the bar chart of the corresponding cell proportions was shown on the right. ns No significant difference. *P* > 0.05; *P < 0.05, **P < 0.01, ***P < 0.001, ****P < 0.0001.

To further observe the effects of RRx-001 on immune cells, we performed immunofluorescence and flow cytometry analysis on tumor tissues. The results showed that after RRx-001 intervention, the expression levels of CD4+ and CD8+ cells in the tumor tissues increased (Fig. 7A, B). In addition, the number of M1-type macrophages increased, the number of M2-type macrophages decreased, and the data of CD8+ Granzyme B and CD8+IFN-γ+ cells increased (Fig. 7C–E). The above results indicate that RRx-001 can exert anti-tumor effects by affecting the function of immune T cells.

## Discussion

The present study elucidates a novel therapeutic mechanism of RRx-001 in HCC, demonstrating its dual role in inducing disulfidptosis—a newly identified form of programmed cell death—and triggering ICD through metabolic-immune crosstalk. While RRx-001 induces both apoptosis and disulfidptosis, the TCEP-reversible component and characteristic F-actin contraction confirm disulfidptosis as the predominant immunogenic death mechanism. Our findings reveal that RRx-001 potently inhibits G6PD, the rate-limiting enzyme of the PPP, leading to NADPH depletion, redox imbalance, and subsequent disulfide stress-mediated cytotoxicity. Importantly, this metabolic disruption not only directly kills HCC cells but also promotes DAMPs (e.g., CRT, HMGB1, HSP70/90) release, reshaping the tumor immune microenvironment toward an anti-tumor phenotype. The differential effects between G6PDi-1 and RRx-001 may stem from their distinct mechanisms of G6PD inhibition and additional immunomodulatory properties of RRx-001, such as ROS/RNS induction. These results position RRx-001 as a promising metabolic-immune modulator for HCC therapy, with potential implications for combination strategies with ICIs.

Disulfidptosis, as a newly discovered type of programmed cell death (PCD), has become a research hotspot in tumor biology. Under nutritional stress conditions in the tumor microenvironment, limited NADPH biosynthesis leads to insufficient reducing equivalents, causing cystine metabolism blockage and disulfide toxicity accumulation. This ultimately triggers disulfidptosis through cytoskeletal protein cross-linking [9]. This metabolically dependent death mechanism provides a theoretical basis for precise intervention strategies based on tumor metabolic vulnerabilities. The PPP, the primary metabolic pathway for intracellular NADPH biosynthesis, dynamically regulates cellular redox homeostasis. Studies have confirmed that glucose deprivation or exogenous oxidative stress (such as H₂O₂ treatment) can effectively induce tumor cell disulfidptosis, but the clinical application is limited [20]. This study innovatively constructs an NADPH metabolic network-targeted intervention system, systematically screening specific inhibitors targeting key metabolic nodes such as folate metabolism, fatty acid oxidation, isocitrate dehydrogenase, glutaminase, NADPH de novo synthesis, and the PPP. By integrating disulfide-specific fluorescent probes with flow cytometry detection technology, we quantitatively evaluated the biological effects of various metabolic intervention schemes on inducing disulfidptosis in HCC models, establishing a precision therapy strategy based on NADPH metabolic regulation. Among the tested inhibitors, only cell apoptosis induced by RRx-001 could be reversed by the disulfide bond-reducing agent TCEP, accompanied by significant F-actin cytoskeleton shrinkage. This characteristic phenotype is highly consistent with disulfidptosis, suggesting that RRx-001 has a specific inductive effect on liver cancer cells.

As a multi-target anticancer drug, RRx-001 exerts its therapeutic effects through various mechanisms, including epigenetic regulation, modulation of the tumor microenvironment, and induction of nitric oxide (NO)-mediated cytotoxicity [21, 22]. Notably, this drug has been proven to be a potent inhibitor of G6PD, a rate-limiting enzyme in the PPP. The activity of G6PD directly impacts the generation of NADPH and redox homeostasis. Studies have revealed that a nanosystem loaded with ozone and RRx-001, upon irradiation, can form reactive nitrogen species (RNS), which enhance immunogenic cell death and promote T-lymphocyte infiltration in triple-negative breast cancer [23, 24]. Additionally, RRx-001 can downregulate PD-L1 expression, increase ROS production, and enhance T-cell infiltration, thereby potentiating immunotherapy in advanced HCC. Furthermore, the combination of RRx-001 and sonodynamic therapy can disrupt redox homeostasis by enhancing immune cell infiltration and inhibiting G6PD expression, ultimately inhibiting the development of osteosarcoma and breast cancer [25, 26]. To delve deeply into the potential underlying mechanism by which RRx-001 induces disulfide death in HCC cells, we conducted a series of detection assays focusing on key metabolites such as the activity of G6PD and the levels of ROS/RNS. The experimental results demonstrated that RRx-001 could significantly inhibit the activity of G6PDH, thereby affecting the PPP, which is a crucial metabolic pathway for the intracellular generation of NADPH. As an important reducing equivalent within cells, NADPH plays a central role in maintaining the intracellular redox balance and the antioxidant defense system. The inhibition of G6PDH activity by RRx-001 led to a reduction in NADPH production, disrupting the original intracellular redox homeostasis. Consequently, ROS/RNS could not be cleared in a timely and effective manner, resulting in their massive accumulation within cells. Excessive ROS/RNS can cause oxidative damage to intracellular biomacromolecules, ultimately triggering disulfide death in HCC cells.

Immunogenic cell death, a specific variant of regulated cell death driven by stress, can induce an adaptive immune response targeting antigens from dying cells. A key feature of immunogenic cell death is the release of DAMPs during the cell death process. These molecules promote T-cell infiltration, converting immunologically “cold” tumors into “hot” ones, thereby enhancing antitumor immune responses. It’s worth noting that in certain contexts, disulfiram/copper death may occur concomitantly with immunogenic cell death [27, 28]. Our findings indicate that when RRx-001 is used to induce disulfidptosis in HCC cells, there is a significant increase in the expression levels of proteins related to DAMPs. This is accompanied by an elevation in tumor CD4+ and CD8 + T-cell levels and a reduction in PD-L1 expression. These observations suggest that during the process of disulfidptosis, cells may release immunostimulatory molecules that trigger immunogenic cell death, providing new perspectives and insights into the mechanisms of cell death in cancer therapy.

In this study, RRx-001 demonstrated significant immunomodulatory effects by inducing disulfidptosis and ICD, thereby reshaping the immune microenvironment of HCC and providing new insights for combination immunotherapy. Firstly, the release of DAMPs, such as CRT and HMGB1, triggered by RRx-001, is pivotal for activating anti-tumor immunity. These molecules act as “danger signals” to promote the maturation and antigen presentation of dendritic cells (DCs), subsequently initiating T-cell responses. Our data revealed a significant increase in the infiltration of CD4+ and CD8 + T cells within tumors following RRx-001 treatment, with CD8+ T cells exhibiting enhanced cytotoxicity (increased secretion of granzyme B and IFN-γ), indicating an effective reversal of the immunosuppressive state in HCC. Additionally, RRx-001 regulated macrophage polarization, evidenced by an increased proportion of M1 macrophages and a decreased proportion of M2 macrophages, further improving the immune microenvironment. This effect may be related to the accumulation of ROS and metabolic reprogramming induced by RRx-001, as ROS can activate pathways such as NF-κB, promoting the secretion of pro-inflammatory cytokines. Secondly, the downregulation of PD-L1 expression by RRx-001 suggests its potential to overcome resistance to immune ICIs. PD-L1 is a crucial mediator of tumor immune evasion, and NADPH metabolic disorders may reduce its expression by affecting PD-L1 glycosylation or stability. Future studies are needed to further validate whether RRx-001 can transform “cold tumors” into “hot tumors” through dual metabolic-immunological mechanisms, thereby expanding the beneficiary population of ICIs.

This study officially confirmed for the first time that RRx-001 can induce disulfide death in HCC cells and induce immunogenic cell death (ICD) by regulating the related pathways of G6PD, NADPH and ROS. This discovery has constructed a bridge connecting NADPH metabolic imbalance with disulfide death and ICD, revealing the significant role of RRx-001 in the treatment of HCC. Unlike the previously known induction methods of disulfide death, this study found that RRx-001 can induce disulfide death in HCC cells under conventional treatment conditions. This discovery provides an important theoretical basis for the clinical transformation and application of RRx-001. Therefore, this study not only reveals the potential of RRx-001 as a novel therapeutic strategy for HCC but also holds significant clinical translational value, offering new perspectives for HCC diagnosis, prognostic evaluation, and therapeutic development. Clinically, the application of RRx-001 may bring new treatment hopes for HCC patients. By quantifying the expression levels of G6PD or the metabolic status of NADPH in tumor tissues, patients can be stratified into different subgroups based on their treatment responses. HCC patients with higher G6PD activity or sufficient NADPH production may respond poorly to RRx-001 treatment, whereas those with reduced G6PD activity or limited NADPH production may benefit more from RRx-001 therapy. Furthermore, the mechanisms of disulfidptosis and ICD induced by RRx-001 provide new ideas for developing precision oncology therapies. Enhancing the pharmacological activity of RRx-001, such as through the development of nanoformulations of RRx-001 or in combination with other immunomodulators, may further improve its anti-tumor effects. Conversely, developing antibody-based targeted therapies against key molecules in the process of disulfidptosis and ICD, such as DAMP release, could also make treatment more precise and effective.

Although this study demonstrates the role of RRx-001 in triggering disulfidptosis via the G6PD-NADPH axis, several limitations remain. The mechanistic depth is still limited, with downstream signaling pathways incompletely elucidated. While our data with TCEP support the link between NADPH depletion and disulfidptosis, future rescue experiments employing NADPH precursors are warranted to provide direct causal evidence. Furthermore, comprehensive time-course experiments will be essential to delineate the precise kinetic relationship between NADPH depletion, ROS accumulation, F-actin contraction, and the execution of disulfidptosis. Furthermore, the reliance on subcutaneous xenograft models may not fully capture the complexity of the tumor microenvironment. Future work should employ orthotopic or humanized mouse models, utilize proteomics and other techniques to unravel molecular details, and explore combination therapies to validate the clinical translational value of RRx-001.

## Conclusion

In summary, RRx-001 represents a first-in-class metabolic-immune therapy for HCC, uniquely inducing disulfidptosis while stimulating anti-tumor immunity. By targeting the G6PD-NADPH axis, it exploits a metabolic Achilles’ heel in HCC while overcoming immunosuppressive barriers. Our work not only advances the mechanistic understanding of disulfidptosis but also opens new avenues for combination immunotherapy in HCC.

## Materials and methods

### Chemicals and reagents

Apoptosis inhibitor, Z-VAD-FMK (MCE). Necrosis inhibitor, Necrostatin-1 (MCE). Ferroptosis inhibitor, Ferrostatin-1. (MCE) Enzyme inhibitors, G6PDi-1 (MCE) and Deferoxaminemesylate (MCE). Other specific target inhibitors include DS18561882(AmBeed), NADS (MCE), AG1-6780(AmBeed), Etomoxir(AmBeed), TCEP (MCE) and RRx-001 (MCE).The following assay kits were acquired from Beyotime Biotechnology: ATP Assay Kit, Glucose-6-Phosphate Dehydrogenase (G6PDH) Activity Assay Kit, Reactive Oxygen Species (ROS) Detection Kit, Glucose Assay Kit, NADP+/NADPH Assay Kit, Reduced/Oxidized Glutathione (GSH/GSSG) Assay Kit, and Nitric Oxide (NO) Detection Kit. Phalloidin was sourced from Solarbio. The following antibodies were obtained from ABclonal Technology: WAVE2/WASF2 Rabbit Monoclonal Antibody (mAb), MYH9 Rabbit Polyclonal Antibody (pAb), ABI2 Rabbit Polyclonal Antibody (pAb), SLC7A11/xCT Rabbit Polyclonal Antibody (pAb), and Vinculin Rabbit Monoclonal Antibody (mAb).

### Cell culture

The hepatocellular carcinoma cell lines, Huh-7 and Hepa1-6, utilized in the TCEP experiments were procured from the Shanghai Cell Bank of the Chinese Academy of Sciences. These cell lines were authenticated via STR profiling and confirmed to be mycoplasma-free. The cells were maintained in DMEM supplemented with 10% FBS and 1% penicillin-streptomycin and incubated at 37 °C in a 5% CO₂ atmosphere. The medium was replaced every 2 days, and the cells were passaged every 3–4 days.

### CCK8 assay

Huh-7 and Hepa1-6 cells in the logarithmic growth phase and exhibiting good growth status were selected. These cells were seeded at a density of 5 × 10^3^ cells per well in a 96-well plate and incubated for 24 h. The medium was then replaced with fresh medium containing various concentrations of different drugs, and the cells were incubated for another 24 h. After the incubation period, 10 µL of CCK-8 reagent was added to each well, and the plate was incubated for 2 h in the dark. The absorbance values (OD values) at a wavelength of 450 nm were measured to determine cell viability. The half-maximal inhibitory concentration (IC50) of each drug was calculated, and a drug concentration of 2×IC50 was chosen for subsequent experimental interventions.

### Flow cytometry for apoptosis detection

To assess the apoptotic rate of cells and evaluate the reversal effect of disulfidptosis inhibitors on apoptosis, flow cytometry was employed. Cells were seeded at a density of 1 × 10^4^ cells per well in a 6-well plate and incubated for 24 h. Following incubation, the medium was replaced with fresh medium containing various drugs for another 24-h intervention. After treatment, the cells were collected, washed twice with pre-cooled PBS, and then stained with Annexin V-FITC and PI. The stained cells were incubated in the dark for 15–30 min before being analyzed by flow cytometry. The apoptotic rate was calculated by analyzing the proportion of cells in different quadrants, and changes in the apoptotic rate before and after treatment with disulfidptosis inhibitors (such as TCEP) were compared.

### Kit assays

To evaluate changes in intracellular disulfidptosis-related markers, various kit assays were utilized, including ATP, GSH/GSSG, NADP+/NADPH, ROS, and NO detection kits. Cells were seeded at a density of 1 × 10^4^ cells per well in a 6-well plate and incubated for 24 h. After incubation, the medium was replaced with fresh medium containing RRx-001 for a specified duration. Following treatment, cells or cell supernatants were collected, and the assays were performed according to the manufacturer’s instructions. The content or activity of each marker was calculated by measuring absorbance values or fluorescence intensity, and differences between treatment groups were compared.

### Transmission electron microscopy

Cells were seeded at a density of 1 × 10^4^ cells per well in a 6-well plate and incubated for 24 h. The medium was then replaced with fresh medium containing low, medium, and high concentrations of RRx-001 for a 24-h treatment. After treatment, the cells were collected, washed twice with pre-cooled PBS, and fixed overnight with 2.5% glutaraldehyde. The next day, the cells were washed three times with PBS for 10 min each, post-fixed with 1% osmium tetroxide for 1 h, and then dehydrated with a graded series of ethanol and acetone. The cells were embedded in epoxy resin, and ultrathin sections were cut using an ultramicrotome after the embedding blocks had solidified. The sections were stained with uranyl acetate and lead citrate and observed under a transmission electron microscope. Images were taken to record ultrastructural changes in the cells.

### Immunofluorescence

Cells were seeded at a density of 1 × 10^4^ cells per well in a 6-well plate and incubated for 24 h. The medium was replaced with fresh medium containing low, medium, and high concentrations of RRx-001 for a 24-h treatment. After treatment, the cells were washed twice with pre-cooled PBS and fixed with 4% paraformaldehyde for 15 min. The cells were then washed three times with PBS for 5 min each, permeabilized with 0.5% Triton X-100 for 10 min, and blocked with 2% BSA for 30 min. Following blocking, the cells were incubated with primary antibodies at 4 °C overnight. The next day, the cells were washed three times with PBS for 10 min each and incubated with fluorescently labeled secondary antibodies at room temperature for 1 h. After washing, the cell nuclei were counterstained with DAPI for 5 min. Finally, the cells were mounted with an anti-fade mounting medium and observed under a fluorescence microscope. Images were taken to record changes in the expression of DAMPs-related markers.

### Metabolomics analysis

Hepa1-6 cells were treated with RRx-001, and a control group was set up. Metabolite samples were obtained from the cells and pretreated. That is, 500 μL of water and 100 μL of methanol were added to each sample, vortices were mixed evenly, and then ultrasonicated for 30 min. After that, the samples were centrifuged at 4 °C and 12,000 RPM for 5 min. Take 20 μL of the supernatant and add 800 μL of acetonitrile. Vortex to mix well and then centrifuge for 5 min at 4 °C and 12,000 RPM. After passing through a 0.22 μm filter, it is ready for machine analysis. Subsequently, the metabolites in the samples were separated and detected using the liquid chromatography-mass spectrometry (LC-MS) analysis platform to obtain the mass spectrometry data of the metabolites. At the same time, prepare standard solutions of different concentrations, obtain the mass spectrometry peak intensity data of the quantitative signals corresponding to each concentration of the standard, and plot the standard curves of different substances with the concentration of the standard as the abscissa and the peak area of the standard as the ordinate. Substitute the ratio of the integral peak areas of all detected samples into the linear equation of the standard curve for calculation. Further substitute the calculation formula to obtain the content data of this substance in the actual sample. Finally, PCA and OPLS-DA analyses were performed using the R. ROPLS software package. The stability of the model was evaluated through 7-period interactive verification, and significant differential metabolites were determined based on VIP > 1 and *p* < 0.05.

### Animal experiments and tumor tissue analysis

Evaluation of the Antitumor Efficacy of RRx-001 In Vivo Using a Subcutaneous Hepatocellular Carcinoma Xenograft Mouse Model. To evaluate the antitumor effects of RRx-001 in vivo, a subcutaneous HCC xenograft mouse model was established. Male C57BL/6 J mice (4–5 weeks old, weighing 15–20 g) were obtained from the Animal Center of Guangxi Medical University. All animal experiments were approved by the Institutional Animal Care and Use Committee of Guangxi Medical University (Approval No. KY-2022-275). Hepa1-6 cells (1 × 10⁶) were subcutaneously inoculated into the right dorsal flank of C57BL/6J mice. When tumor volumes reached approximately 100 mm³, the mice were randomly divided into a control group and an RRx-001 treatment group (*n* = 6 per group) using a digital randomization method. Tumor volume measurements and immunohistochemical analyses were conducted by investigators blinded to group allocation. RRx-001 was administered via intraperitoneal injection (5 mg/kg) every other day for a total of five doses, followed by a 1-week observation period. Throughout the treatment, the survival status of the mice was monitored, and tumor length and width were measured using vernier calipers to calculate tumor volume (volume = 1/2 × length × width²).

Upon completion of the treatment, the mice were euthanized, and tumor tissues were harvested. The excised tumors were divided into three parts for subsequent analyses: flow cytometry, immunohistochemistry (IHC), and whole-transcriptome sequencing. Wash the tumor tissue with pre-cooled PBS, cut it into small pieces, and add collagenase and DNase. Shake and digest at 37 °C. Then, pass the tissue through a 200-mesh sieve to obtain a single-cell suspension. Centrifuge and discard the supernatant. Take an appropriate amount of the cell suspension, add anti-CD16/32 antibody to block the Fc segment to reduce non-specific binding, then add anti-CD45, F4/80, and CD8 antibodies, as well as antibodies labeled M1 (such as CD86) and M2 (such as CD206) macrophages, respectively, and incubate at 4 °C in the dark for 30 min. When detecting CD8+ Granzyme B and CD8+IFN-γ+ cells, after fixation and membrane-breaking treatment, the corresponding antibodies were added and incubated at 4 °C in the dark for 30 min. After staining was completed, the cells were washed with PBS, resuspended in buffer, and detected by flow cytometry. By setting appropriate gating strategies, the proportions of M1- and M2-type macrophages in tumor tissues, as well as the proportions of CD8+ Granzyme B and CD8+IFN-γ+ cells in CD8+ T cells, were analyzed.

For IHC analysis, tumor tissues were fixed in 4% paraformaldehyde, embedded in paraffin, and sectioned. The sections underwent deparaffinization, antigen retrieval, blocking, incubation with primary antibodies, secondary antibody incubation, and 3,3′-diaminobenzidine (DAB) staining. Protein expression changes were then visualized and documented under a microscope. For whole-transcriptome sequencing, total RNA was extracted from tumor tissues of the control and treatment groups using TRIzol reagent. RNA quality and concentration were assessed via agarose gel electrophoresis and NanoDrop spectrophotometry. Qualified RNA samples were used to construct sequencing libraries, followed by Illumina sequencing. After quality control, bioinformatics tools were employed to identify differentially expressed genes (DEGs), perform Gene Ontology (GO) enrichment analysis, and conduct Kyoto Encyclopedia of Genes and Genomes (KEGG) pathway analysis to untangle the impact of RRx-001 treatment on the tumor transcriptome.

### Western blotting

Cells (1 × 10⁴) were seeded into 6-well plates and incubated for 24 h before being treated with media containing RRx-001 or inhibitors for another 24 h. Following treatment, cells were collected, and total protein was extracted. Protein concentration was determined using the BCA method. Subsequently, equal amounts of protein were separated by SDS-PAGE electrophoresis and transferred to a PVDF membrane. After blocking with 5% skim milk for 1 h, the membrane was incubated with primary antibodies at 4 °C for 12 h. It was then washed three times with TBST for 10 min each, followed by incubation with secondary antibodies at room temperature for 1 h. After additional washes, the membrane was developed using an ECL chemiluminescence kit and photographed in a gel imaging system. Changes in protein expression levels between different treatment groups were compared through grayscale value analysis.

### Statistical analysis

Data analysis was performed using ImageJ, FlowJo, SPSS 27.0 (version 27.0.0.0), and GraphPad Prism 9.5 (version 9.5.0). Normality of data distribution was assessed using Shapiro-Wilk test, and homogeneity of variance was evaluated using Levene’s test. Measurement data were expressed as mean ± standard deviation (mean ± SD). Comparisons between two groups were made using an independent sample *t*-test, while comparisons among multiple groups were analyzed using one-way ANOVA, followed by Tukey’s test for multiple comparisons. Statistical significance was set at *P* < 0.05. All cell experiments were repeated three times.

## Supplementary information

Supplementary materials Western blot

Supplementary figure

## Data Availability

The data that support the findings of this study are available from the corresponding author upon reasonable request.
