# Supplementary material for: RRx-001 inhibits G6PD to deplete NADPH and trigger disulfidptosis coupled with DAMP-mediated immunogenic cell death in hepatocellular carcinoma
Source: Cell Death Discov. 2026 Mar 26;12:194. doi: 10.1038/s41420-026-03032-y (PMC13144330; doi:10.1038/s41420-026-03032-y)
Supplement: Supplementary file 1 — Supplementary materials Western blot [file 41420_2026_3032_MOESM1_ESM.pdf]

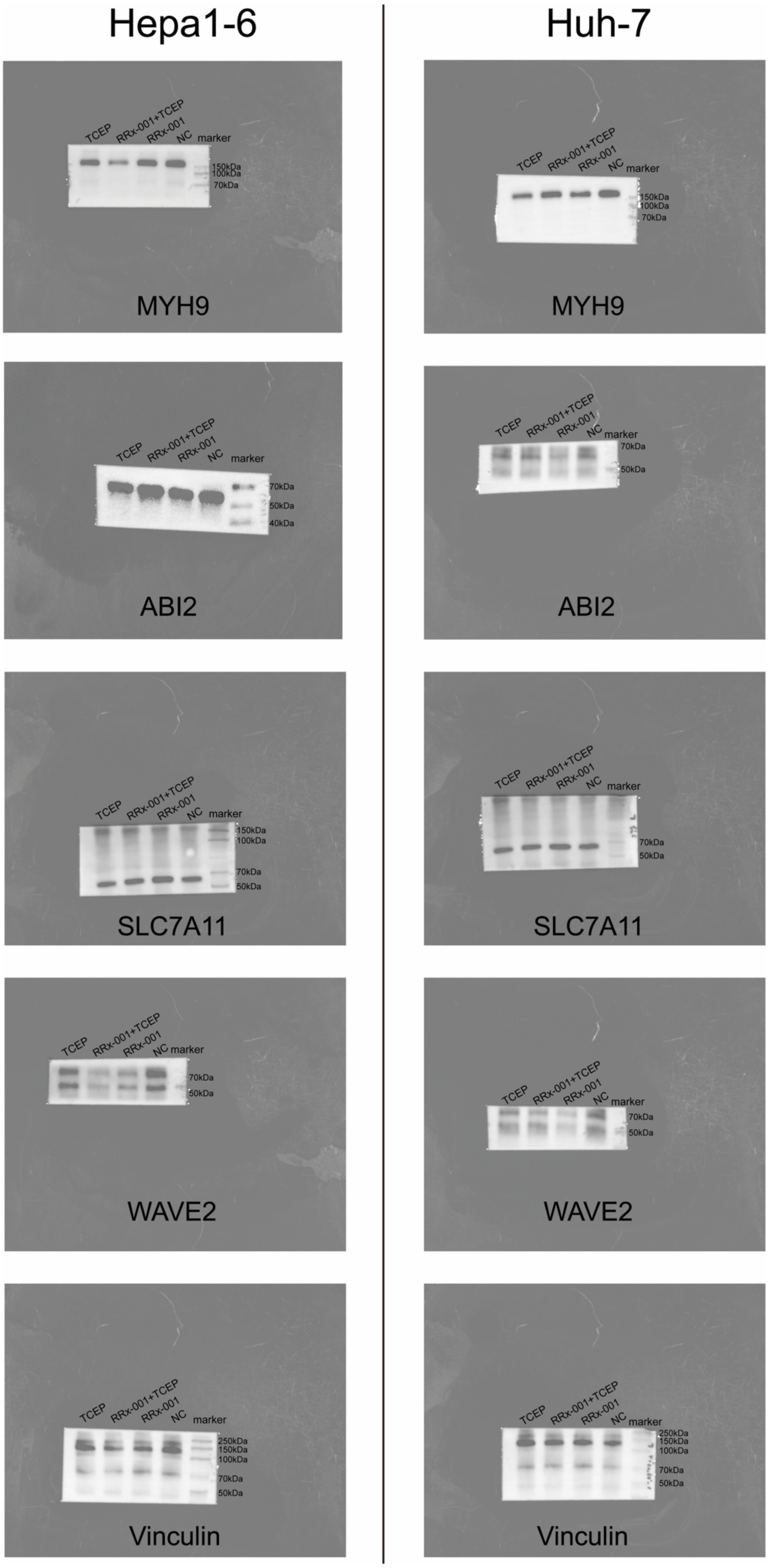

Figure S6: Supplementary materials Western blot 1

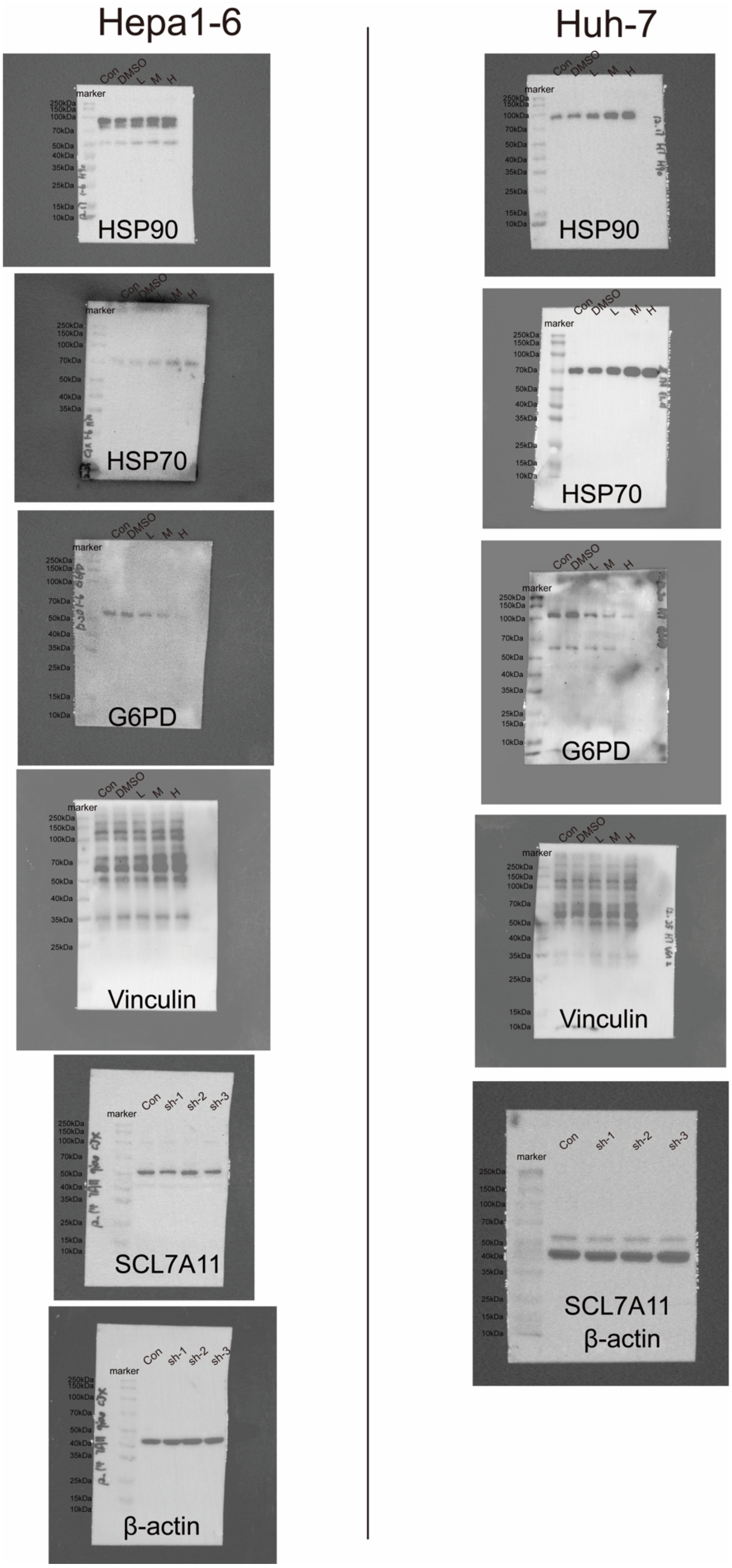

Figure S7: Supplementary materials Western blot 2
