# Supplementary material for: RRx-001 inhibits G6PD to deplete NADPH and trigger disulfidptosis coupled with DAMP-mediated immunogenic cell death in hepatocellular carcinoma
Source: Cell Death Discov. 2026 Mar 26;12:194. doi: 10.1038/s41420-026-03032-y (PMC13144330; doi:10.1038/s41420-026-03032-y)
Supplement: Supplementary file 2 — Supplementary figure [file 41420_2026_3032_MOESM2_ESM.pdf]

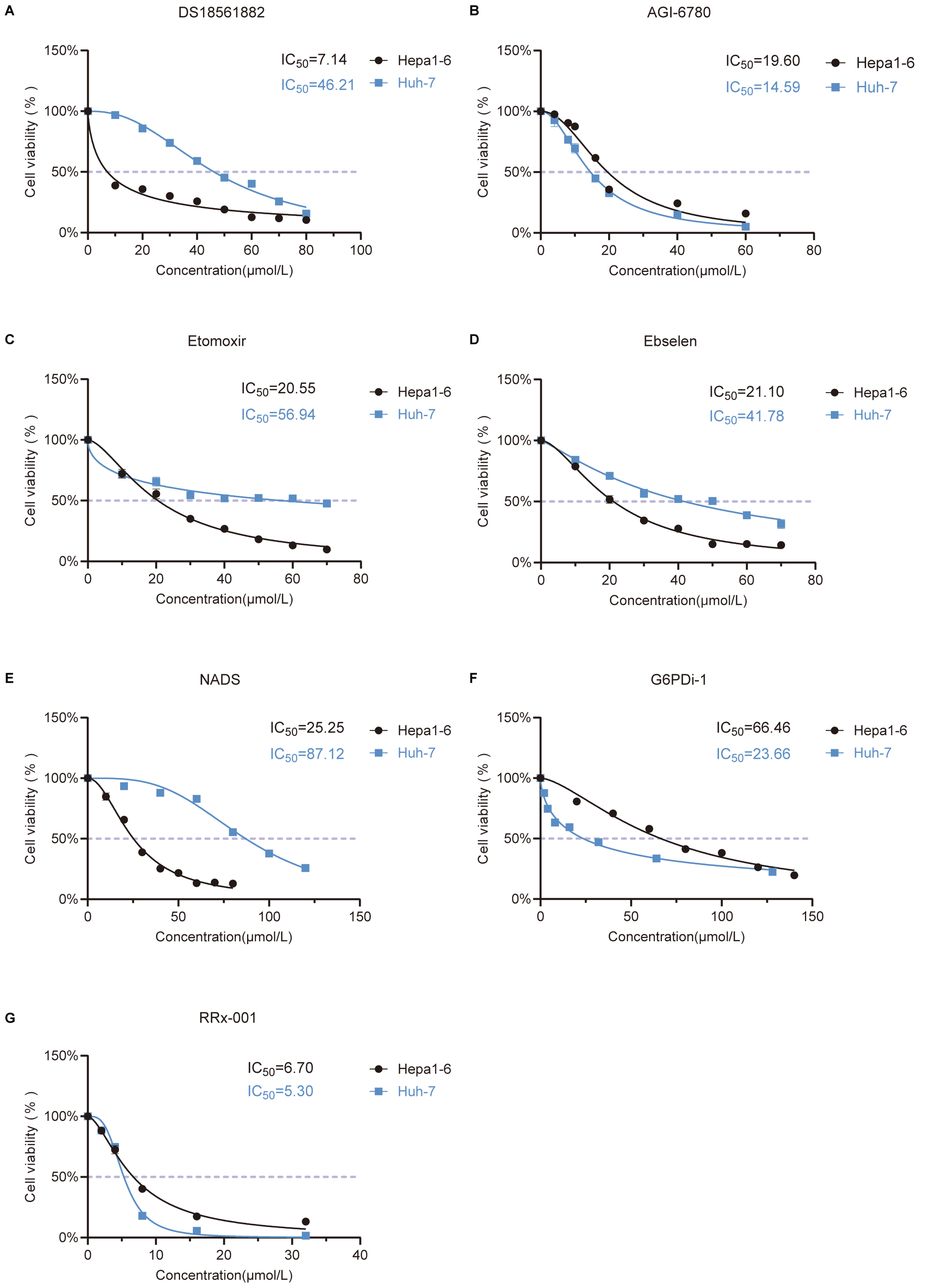

Figure S1 The effect of NADPH inhibitors on the viability of Huh-7 and Hepa1-6 cells. A-G represents the sensitivity of Huh-7 and Hepa1-6 cells to seven NADPH inhibitors, namely DS18561882, AGI-6780, Etomoxir, Ebselen, NADS, G6PDi-1, and RRx-001, respectively.

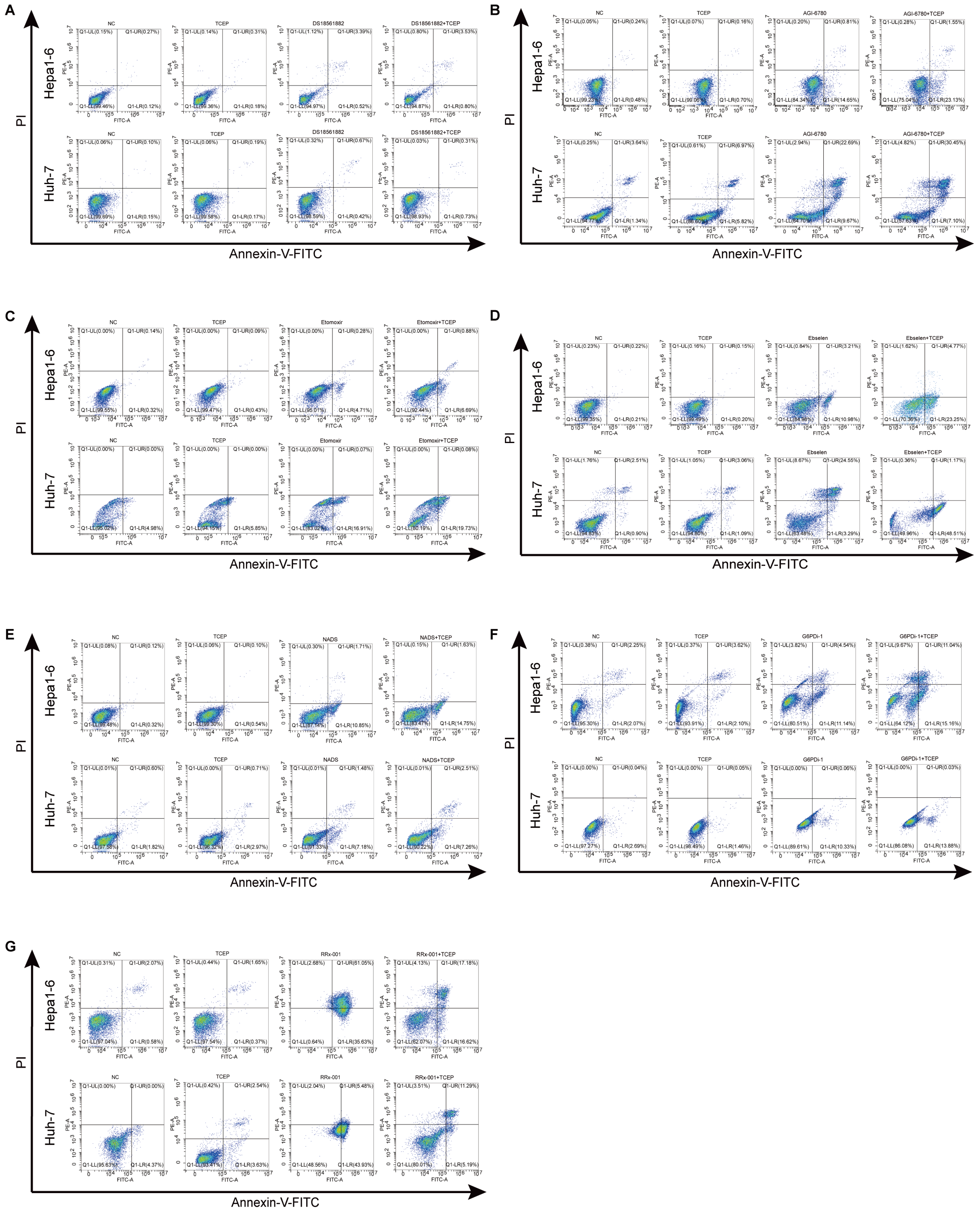

Figure S2 The effect of NADPH inhibitors on the apoptosis of Huh-7 and Hepa1-6 cells. A-G represents the apoptosis detection of Huh-7 and Hepa1-6 cells against seven NADPH inhibitors, namely DS18561882, AGI-6780, Etomoxir, Ebselen, NADS, G6PDi-1 and RRx-001.

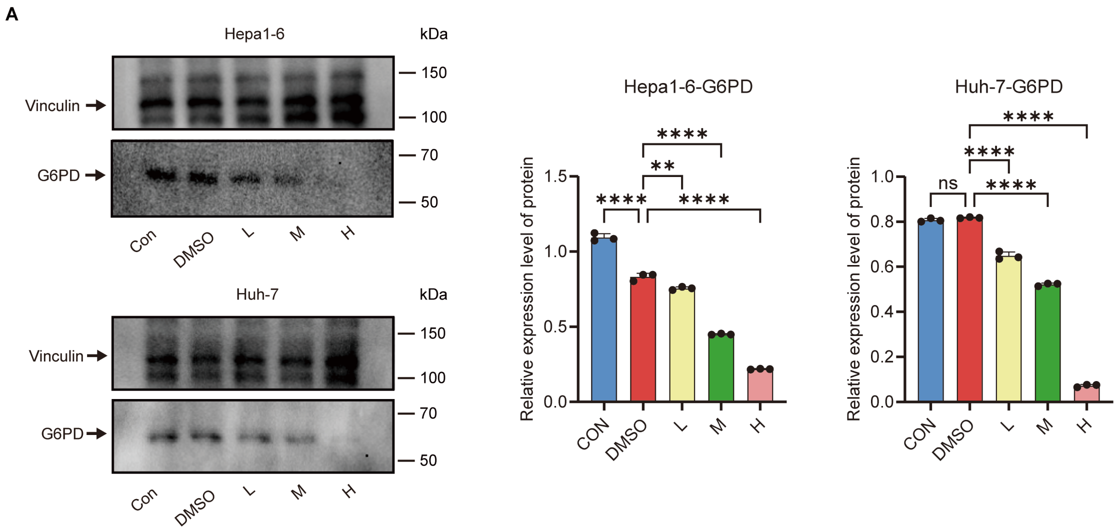

Figure S3 Western blot analysis of G6PD expression in HCC cells treated with RRx-001. ns, No significant difference. P>0.05; *P<0.05, **P<0.01, ***P<0.001, ****P<0.0001.

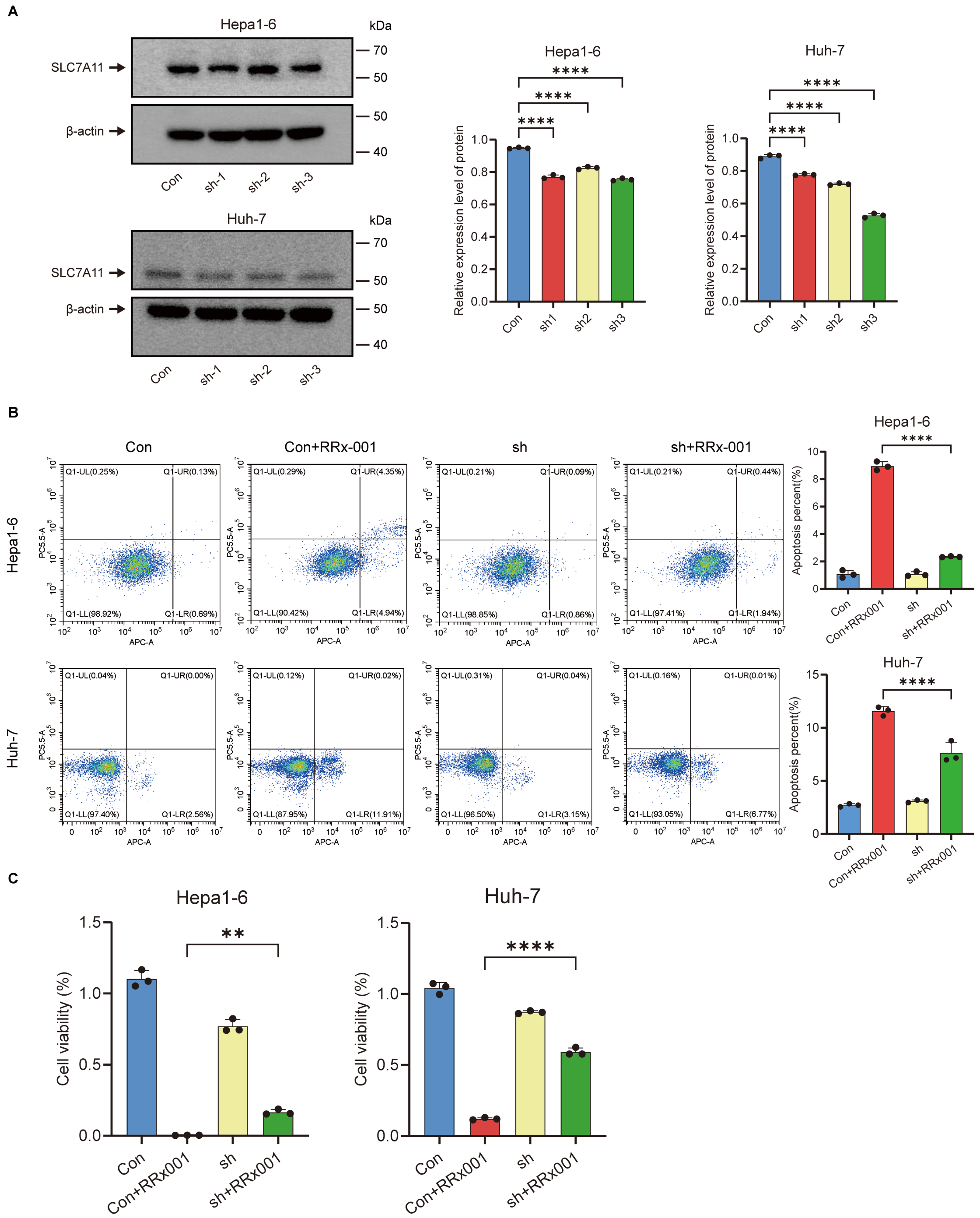

Figure S4 Effect of SLC7A11 knockdown on RRx-001-induced cell death. A, After transfection of shRNA into Hepa1-6 and Huh-7 cells, the knockout efficiency of SLC7A11 was verified by western blotting. B, After selecting the most effective knockdown efficiency sites, the RRx-001 drug was added for cultivation. Flow cytometry was used to observe the apoptosis rates of the negative control group and the knockdown group. C, Add the RRx-001 drug culture, and use the CCK8 method to observe the cell survival rates of the negative control group and the knockdown group.

ns, No significant difference. P>0.05; *P<0.05, **P<0.01, ***P<0.001, ****P<0.0001.

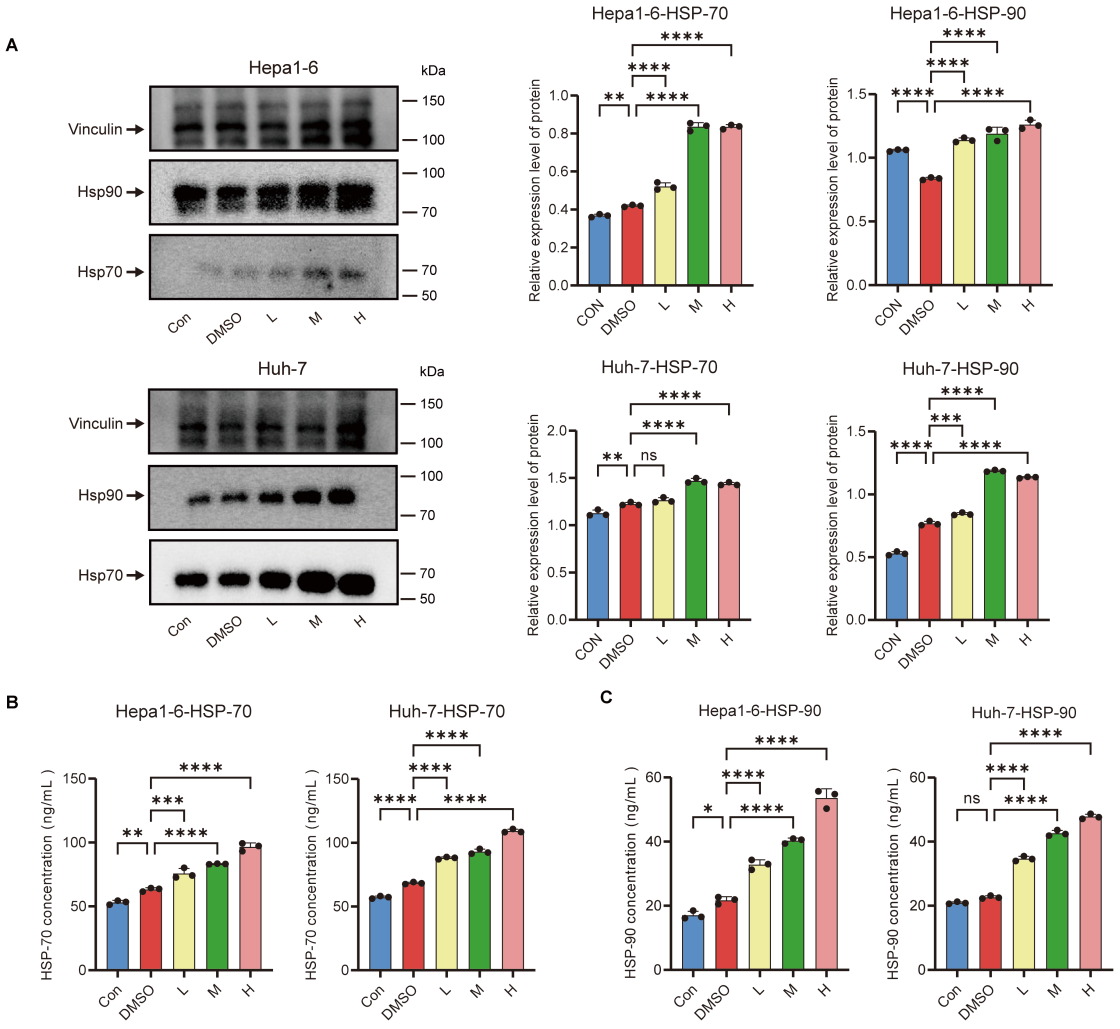

Figure S5 Western blot and ELISA detection of extracellular HSP70 and HSP90 release. A, After adding the RRx-001 drug, western blot was used to observe the expression of HSP70 and HSP90. B-C, After adding the RRx-001 drug, the expression of HSP70 and HSP90 was observed using the Elisa method. ns, No significant difference. P>0.05; *P<0.05, **P<0.01, ***P<0.001, ****P<0.0001.
